# CRISPR/Cas9-mediated *AtGATA25* mutant represents a novel model for regulating hypocotyl elongation in *Arabidopsis thaliana*

**DOI:** 10.1007/s11033-022-07926-9

**Published:** 2022-10-27

**Authors:** Kihwan Kim, Juhyung Shin, Tae-An Kang, Byeonggyu Kim, Won-Chan Kim

**Affiliations:** 1 Department of Applied Biosciences, Kyungpook National University, 41566 Daegu, Republic of Korea; 2 Department of Integrative Biology, Kyungpook National University, 41566 Daegu, Republic of Korea

**Keywords:** AtGATA25, Circadian clock, CRISPR/Cas9, Hypocotyl elongation, Phytochrome interacting factor 4

## Abstract

**Background:**

Plants have evolved to adapt to the ever-changing environments through various morphological changes. An organism anticipates and responds to changes in its environment via the circadian clock, an endogenous oscillator lasting approximately 24 h. The circadian clock regulates various physiological processes, such as hypocotyl elongation in *Arabidopsis thaliana*. Phytochrome interacting factor 4 (PIF4), a member of the bHLH protein family, plays a vital hub role in light signaling pathways and temperature-mediated growth response mechanisms. PIF4 is controlled by the circadian clock and interacts with several factors. However, the components that regulate PIF4 transcription and activity are not clearly understood.

**Methods and Results:**

Here, we showed that the *Arabidopsis thaliana GATA25* (*AtGATA25*) transcription factor plays a fundamental role in promoting hypocotyl elongation by positively regulating the expression of PIF4. This was confirmed to in the loss-of-function mutant of AtGATA25 via CRISPR/Cas9-mediated gene editing, which inhibits hypocotyl elongation and decreases the expression of *PIF4*. In contrast, the overexpression of *AtGATA25* in transgenic plants resulted in increased expression of *PIF4* and enhanced hypocotyl elongation. To better understand AtGATA25-mediated PIF4 transcriptional regulation, we analyzed the promoter region of the target gene *PIF4* and characterized the role of GATA25 through transcriptional activation analysis.

**Conclusion:**

Our findings suggest a novel role of the AtGATA25 transcription factor in hypocotyl elongation.

**Supplementary Information:**

The online version contains supplementary material available at 10.1007/s11033-022-07926-9.

## Introduction

Plants as sessile organisms must be in harmony with vital environmental factors that regulate physiological processes such as light and temperature for efficient survival and reproduction [1, 2]. Key environmental factors provide a very complex signaling mechanism that accompanies the circadian rhythm of the Earth’s 24-hour rotation [3]. Many molecular biological studies have shown that at least one-third of the genes in *Arabidopsis thaliana* are expressed according to the circadian rhythm [4–6]. The circadian rhythm is regulated by the oscillator component, which is the late elongated hypocotyl (LHY), the circadian clock associated 1 (CCA1), and the pseudo-responsive regulatory protein, the timing of cab expression 1 (TOC1) [7–9]. The mechanism of regulation by several circadian clock components is that the circadian clock and light signals mediate hypocotyl growth regulation [10, 11]. Furthermore, five phytochrome and two cryptochrome photoreceptors in *Arabidopsis thaliana* transduce various light signals into downstream light signal components, thereby playing a role in initiating diverse cellular and physiological processes [12, 13].

Phytochrome interacting factor 4 (PIF4), a bHLH transcription factor, controls hypocotyl growth by converging on the PIF4-mediated auxin signaling pathway through multiple signaling cascades, such as the circadian clock, light signaling, and temperature [14–16]. Thus, PIF4 plays a vital role in governing seedling growth as a central hub for several signaling pathways. Particularly, it has been reported that PIF4 regulates the expression of sub-genes according to the plant circadian clock through physical binding with the TOC1 protein [17]. A previous study reported that TCP17 positively modulated PIF4 activity [18]. However, given the fact that many factors have been proposed to interact with and inhibit PIF4 activity, it is thought that the regulatory components that positively regulate PIF4 activity are thought to vary. GATA transcription factors are involved in light-mediated and circadian regulation gene expression [19, 20]. There are 29 types of *GATA* genes in *Arabidopsis thaliana* genome, and they have C-Χ_2_-C-Χ_18_-C-Χ_2_-C or C-Χ_2_-C-Χ_20_-C-Χ_2_-C conserved cysteine residues of the zinc-finger domain. Each GATA transcription factor is divided into four types of sub-family according to the additional motif. In this study, we attempted to confirm the characteristics of *Arabidopsis thaliana GATA25* (*AtGATA25*), also known as ZIM, which belongs to sub-family III and has the C-Χ_2_-C-Χ_20_-C-Χ_2_-C of the zinc-finger domain. The overexpression of *AtGATA25* (*ZIM*) in *Arabidopsis thaliana* was reported to induce hypocotyl elongation under red, far infrared, blue, and white light [21]. Previous studies have suggested that AtGATA25 is under strong circadian regulation [22] and that transcription factors influence the post-integration of photoreceptor-mediated light signal transduction. However, the understanding of the molecular mechanism according to the light signal is insufficient. We attempted to present a novel regulatory mechanism for regulating hypocotyl elongation of *AtGATA25* (ZIM), which is known in previous studies to regulate cell elongation [21]. Therefore, we tried to confirm the role of AtGATA25 according to the light signal through the analysis of *gata25* mutant using the CRISPR/Cas9-mediated gene editing system.

The function of a gene can be most efficiently confirmed by introducing nucleotide insertions, deletions, or substitutions into the sequence of the target gene (knockout) using genome editing technology that uses sequence-specific nucleases (SSN) [23]. The introduction of SSN-mediated genome editing tools in plants has led to many advances in biology, including plant genetic engineering. Three types of SSN systems, zinc-finger nucleases (ZFNs), transcription activator-like effector nucleases (TALENs), and clustered regularly interspaced short palindromic repeats (CRISPR)/CRISPR-associated system 9 (Cas9), were used for genetic modification [24–26]. Among these three types of SSN systems, the CRISPR/Cas9 system showed design flexibility, versatility, and high efficiency in *Arabidopsis* [27], maize [28], rice [29], wheat [30], and tomato [31]. Three components of CRISPR/Cas9-mediated DNA recognition and cleavage exist: Cas nucleases, CRISPR RNA (crRNA), and trans-activating crRNA (tracrRNA). The Cas9 protein achieves target site cleavage after recognizing the NGG protospacer adjacent motif (PAM) adjacent to the 3′ end of the target sequence of small-guide RNA (sgRNA), a complex of tracrRNA and crRNA. CRISPR/Cas9-induced site-specific DNA double-strand breaks (DBS) result in nucleotide insertions, deletions, or substitutions in gene-coding regions via homology-directed repair (HDR) or non-homologous end-joining (NHEJ) pathways.

Here, we generated *gata25* loss-of-function mutants in which hypocotyl elongation was inhibited regardless of the length of the day and night. Furthermore, our molecular biology studies showed that AtGATA25 positively regulates hypocotyl elongation by binding to the promoter of *PIF4* and upregulating its transcription to maintain proper expression. Therefore, our study presents a novel molecular mechanism that regulates hypocotyl growth via PIF4.

## Materials and methods

### Plant materials and growth conditions

*Arabidopsis thaliana*, ecotype Columbia (Col-0) was used throughout this study. Wild-type and mutant seeds were treated in the 0.5 ⋅ Murashige and Skoog (^1^/_2_ MS) medium [32] containing 1% (w/v) sucrose, and 0.6% (w/v) phytoagar in the dark conditions at 4℃ for 3 days before germination. Plants were grown on ^1^/_2_ MS agar plates in a growth chamber at 23℃ under long-day conditions (LD, 16 h light/8 h dark photoperiod) or under short-day conditions (SD, 8 h light/16 h dark photoperiod). The light intensity was approximately 100 µmol m^− 2^s^− 1^.

### Construction of vectors and the generation of *AtGATA25* overexpression transgenic plants

To generate transgenic plants overexpressing of *AtGATA25* (At4g24470), full-length GATA25 CDS was amplified from *Arabidopsis* leaf cDNA by polymerase chain reaction (PCR) using primers linked to *Spe*I and *Xma*I sites (shown in lower-case letters): 5’- GGactagtATGTTTGGTCGCCATTC − 3’ (forward) and 5’- CCCcccgggTTAGTGATCACCTAAC − 3’(reverse). The PCR product was cloned into a homemade binary pTK-BMLC vector [33, 34]. The vector construct used in this study was verified by the Sanger sequencing.

The pTK-BMLC binary vector was transformed into the *Agrobacterium tumefaciens* strain GV3101 and used for *Agrobacterium*-mediated transformation of *Arabidopsis thaliana* (Col-0) [35]. Briefly, a single *A. tumefaciens* strain (GV3101) containing the pTK-BMLC binary vector construct was cultured to a final OD_600 nm_ of 0.8 and then suspended in a floral dip inoculation medium containing 5% (w/v) sucrose and 0.05% (w/v) Silwet L-77 (Lehel Seeds, Round Rock, TX, USA). Floral dip inoculation medium was used for the transformation of *Arabidopsis thaliana*, and transgenic plants were sprayed with Basta solution containing 180 mg/ml glufosinate ammonium as an active ingredient and as a selection marker.

### Designing the common gRNA target sequence and constructing the CRISPR/Cas9 vector

The sequence of *AtGATA25* (At4g24470) was obtained from The Arabidopsis Information Resource (TAIR). Sequence analysis of *AtGATA25* genes was conducted using the web tools of CRISPR-P version 2.0 online software (http://crispr.hzau.edu.cn/CRISPR2/) [36]. The common gRNA target sequence was selected in the second exon of *AtGATA25* (GCCTCCGATTTGATTCCCGATGG; the PAM sequence is underlined).

To construct sgRNA modules for gene editing in *Arabidopsis*, we used the pKSE401 vector which is the CRISPR/Cas9 plant expression vector. The vector was constructed by modifying a previously described method [37]. Briefly, equal volumes of 100 µmol/ml *AtGATA25* gRNA_F (attgGCCTCCGATTTGATTCCCGA) and *AtGATA25* gRNA_R (aaacTCGGGAATCAAATCGGAGGC) were mixed and incubated for 5 min at 95℃ and then slowly cooled at 25℃ to produce a double-stranded insert with 4 nucleotide 5’ overhangs. The assembled gRNA as a double-stranded insert was inserted into the pKSE401 vector by following the Golden Gate Assembly [38], using *BsaI* and T4 Ligase (NEB, Ipswich, MA, USA).

### Generation of CRISPR/Cas9-mediated transgenic plants and selection of transgenic plants from T_0_ plants

To generate the *gata25* mutant, the CRISPR/Cas9 binary vector construct was introduced into the *Agrobacterium tumefaciens* strain GV3101 and used by following a modified floral dip method [35].

All the collected candidate transgenic seeds were grown on a medium containing kanamycin (50 µg/ml) to select the T_1_ generation of transgenic plants (T_1_ plants) from the T_0_ generation of transgenic plants (T_0_ plants) [39]. Briefly, the collected T_0_ seeds were sterilized with 70% (v/v) ethanol for 1min, followed by seed disinfectant (25% (v/v) bleach and 0.01% (v/v) Triton X-100) for 5min. After that, the seeds were washed five times with sterile distilled water. The surfaced-sterilized seeds were sown on ^1^/_2_ MS agar plates containing kanamycin (50 µg/ml) and cold-treated seeds in the dark at 4℃ for 3 days. After the cold-treated seeds were transferred to a growth chamber and exposed to continuous white light (100 µmol m^− 2^s^− 1^) for 6 h at 23℃ for germination. The plate with sown seeds was kept in dark condition for 48 h at 23℃. Finally, the seed in the plate was incubated for 24 h at 23℃ to select T_1_ plants with expanded green cotyledons.

### Identification of the *gata25* mutant and validation of CRISPR/Cas9-mediated target mutation

For the identification of *gata25* mutant lines, genomic DNA from young leaves was extracted for use in PCR-amplified reaction according to the preparation of plant genomic DNA [40]. Briefly, the young leaves were ground with a pestle and was added 400 µl of modified Edwards solution (200 mM Tris-HCl, 25 mM EDTA, 1% (w/v) SDS, 400 mM LiCl, pH 9.0), and the sample vortexed. After the mixture sample was centrifuged at 13,000 ⋅ g for 5min, the supernatant was mixed with an equal volume of isopropanol. The sample was centrifuged at 13,000 ⋅ g for 10 min and then the supernatant was discarded. The pellet was washed with 1 ml of 70% ethanol. Following centrifugation at 13,000 ⋅ g for 5 min, the precipitation was dried and dissolved in 20 µl of DNase-free distilled water. The extracted genomic DNA of each transgenic and wild-type plant was subjected to PCR to amplify the surrounding target site using specific primers (Supplementary Table S1). The PCR products were confirmed using the T7 endonuclease I (T7E1) assay [41]. To perform of T7E1 assay, each PCR product was denatured at 95℃ for 5 min. Then, annealed at -2℃ per second with a temperature ramp to 85℃ for 5s and − 0.1℃ per second with a temperature ramp to 25℃ for 10 min. The generated hetero-type DNA was digested with T7E1 enzyme (NEB, Ipswich, MA, USA) at 37℃ for 30 min, then electrophoresed on a 2% (w/v) agarose gel.

For validation of mutations, the PCR was performed to amplify the neighboring on-target region using the genomic DNA of selected homozygous plants, and then the PCR product was analyzed by the Sanger sequencing.

### Evaluation of hypocotyl length

Seedlings that were not in contact with others were photographed using a Leica EZ4E microscope, five days after sowing. The hypocotyl length was measured using using IMT iSolution Lite software (Burnaby, BC, Canada) as the distance from the collet of root hairs to the ‘v’ made by the cotyledon shoulder.

### Quantitative real-time PCR (qRT-PCR)

Total RNAs were isolated by using TRIzol reagent (Invitrogen). The isolated total RNAs were treated with RNase-free DNaseI (Qiagen). First-strand cDNA was synthesized from 1000 ng of total RNA using SuperScript III Reverse Transcriptase (Invitrogen). PowerUp™ SYBR™ Green Master Mix (Thermo Fisher) was used to calculate double-strand DNA synthesis for the real-time PCR reaction. To compare gene transcription levels, the level of *PP2A* mRNA expression (At1g13320) was used as a control. The details of the primers used in this study are provided in Supplementary Table S1.

### Transcriptional activation analysis (TAA)

To construct the effector, the full-length *AtGATA25* CDS was amplified from the leaf cDNA of *Arabidopsis thaliana* by PCR using primers linked to *Spe*I and *Xma*I sites, respectively. The PCR products of *AtGATA25* CDS were ligated between 2Χ CaMV 35S promoter and NOS terminator in pTrTk-BMLC. pTrTk-BMLC is a homemade vector designed such that pTrGUS vector backbone is used to replace the CaMV 35S promoter with a 2Χ CaMV 35S promoter and the *GUS* reporter gene is removed. To construct the reporter, 1,434 bp of the *AtPIF4* promoter was amplified from leaf gDNA of *Arabidopsis thaliana* using primers linked to *Pst*I and *Bam*HI, respectively. The PCR products of the *AtPIF4* promoter were cloned in front of the *GUS* reporter gene into the pTrGUS vector.

Transcriptional activation analysis was performed using protoplasts isolated from *Arabidopsis thaliana* leaves. As previously described, protoplast isolation and polyethylene glycol (PEG)-mediated transformation of reporter and effector constructs were performed [42]. For the transcriptional activation analysis, protoplasts transfected with the effector and reporter were lysed after incubation for 12 h at room temperature. The GUS protein activity was measured by using the SPECTRAmax GEMINI XS microplate spectrofluorometer (Molecular devices) for 4-methylumbelliferone, a degradation product of the reaction.

For the verification of the direct target gene, the construct vector in which the full-length cDNA of *AtGATA25* was fused in front of the glucocorticoid receptor (GR) was used as an effector. To activate AtGATA25, which is fused to the N-terminus of GR, the protoplasts were treated with 10 µM dexamethasone (DEX). To inhibit new protein synthesis, 2 µM of cycloheximide (CHX) was treated 30 min before the addition of DEX [43]. Total RNAs were extracted from the protoplasts after treatment to confirm gene expression levels.

### Statistical analysis

Data are presented as the mean of at least three independent biological replicates, ± standard deviation (SD). Differences were considered statistically significant when the *p* < 0.05 (labeled “*”), *p* < 0.01 (labeled “**”) or *p* < 0.001 (labeled “***”) using a pairwise Student’s *t*-test.

## Results

### Generation of *AtGATA25* knockout plants using the CRISPR/Cas9 system

**Fig. 1:**
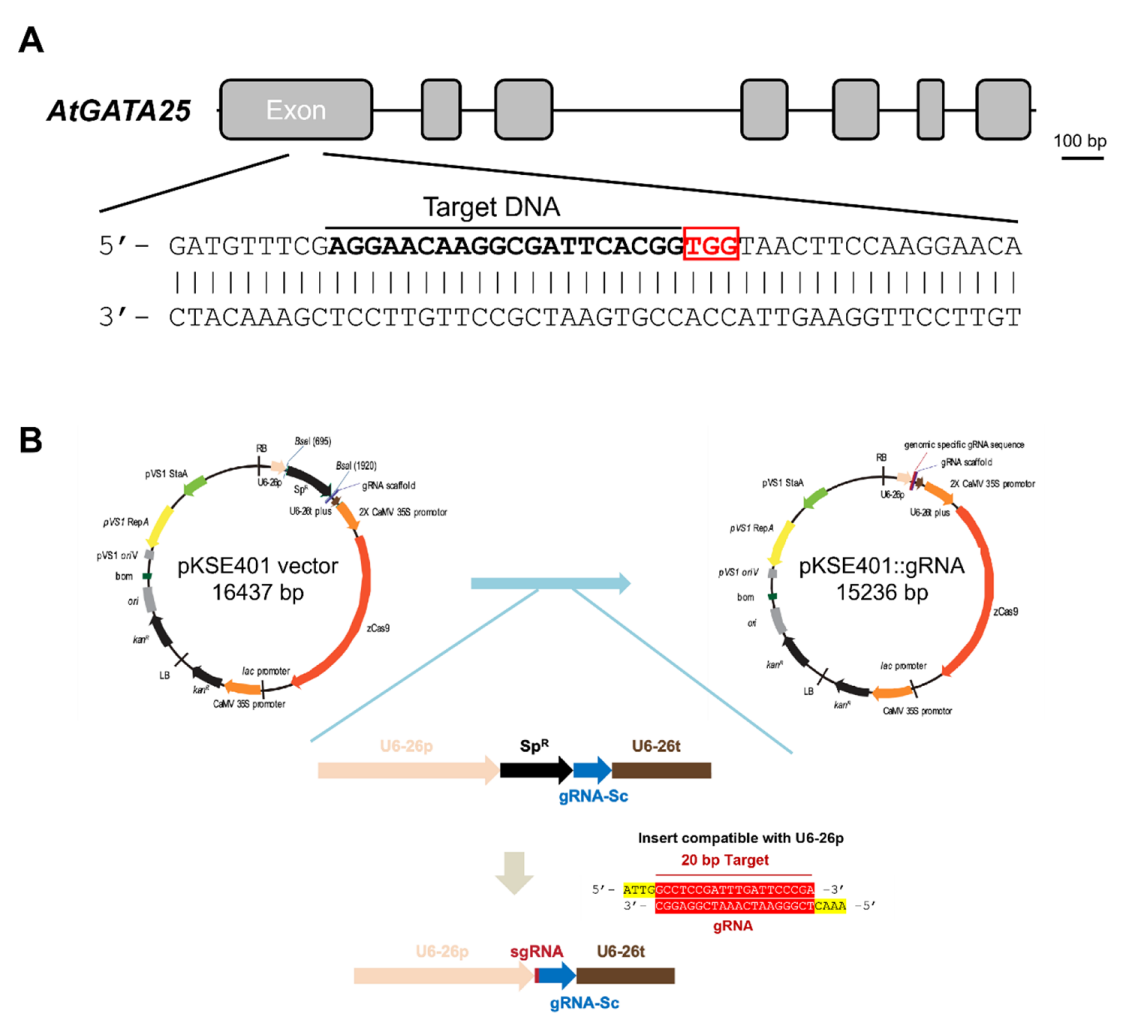
Physical maps and structure of CRISPR/Cas9 binary vector. **A** Schematic representation of *AtGATA25*. **B** Premade gRNA modules used for the assembly of gRNA expression cassettes. The gRNA-targeted DNA are underlined and the protospacer adjacent motif (PAM) is in the red box. Black line, intron; Gray box, exon, Scale bars, 100 bp

To verify the genome structure and sequence of *AtGATA25* in *Arabidopsis thaliana*, primers were designed based on the nucleotide sequence obtained from the TAIR. The *AtGATA25* gene coding sequence was amplified from the leaf cDNA of *Arabidopsis thaliana* Col-0 by PCR using the primers and sequenced. We obtained the 930bp of the AtGATA25 gene sequence, an alternative spliced form of At4g24470.1, instead of the representative gene model, At4g24470.3 annotated at TAIR. Further sequencing of the *AtGATA25* cDNA sample confirmed that *AtGATA25* contains seven exons and six introns and encodes a protein of 309 amino acids (Fig.1a, Supplementary Fig. S1). The first exon of *AtGATA25* encodes an N-terminal 102 amino acid sequences, whereas the zinc-finger domain responsible for the DNA-binding function of the AtGATA25 protein is located in the regions of amino acids 214–248 of the fifth exon. In addition, the third and fourth exons encode the region of 146–190 amino acid residues of the CONSTANS, CO-like, and TOC1 (CCT) domains. Therefore, the first exon was selected to efficiently counteract the function of the AtGATA25 protein. Based on the CRISPR-P version 2.0 online software prediction of *AtGATA25*, sgRNAs with high on-score, low off-score, and high GC content were designed (Supplementary Fig. S2).

**Fig. 2:**
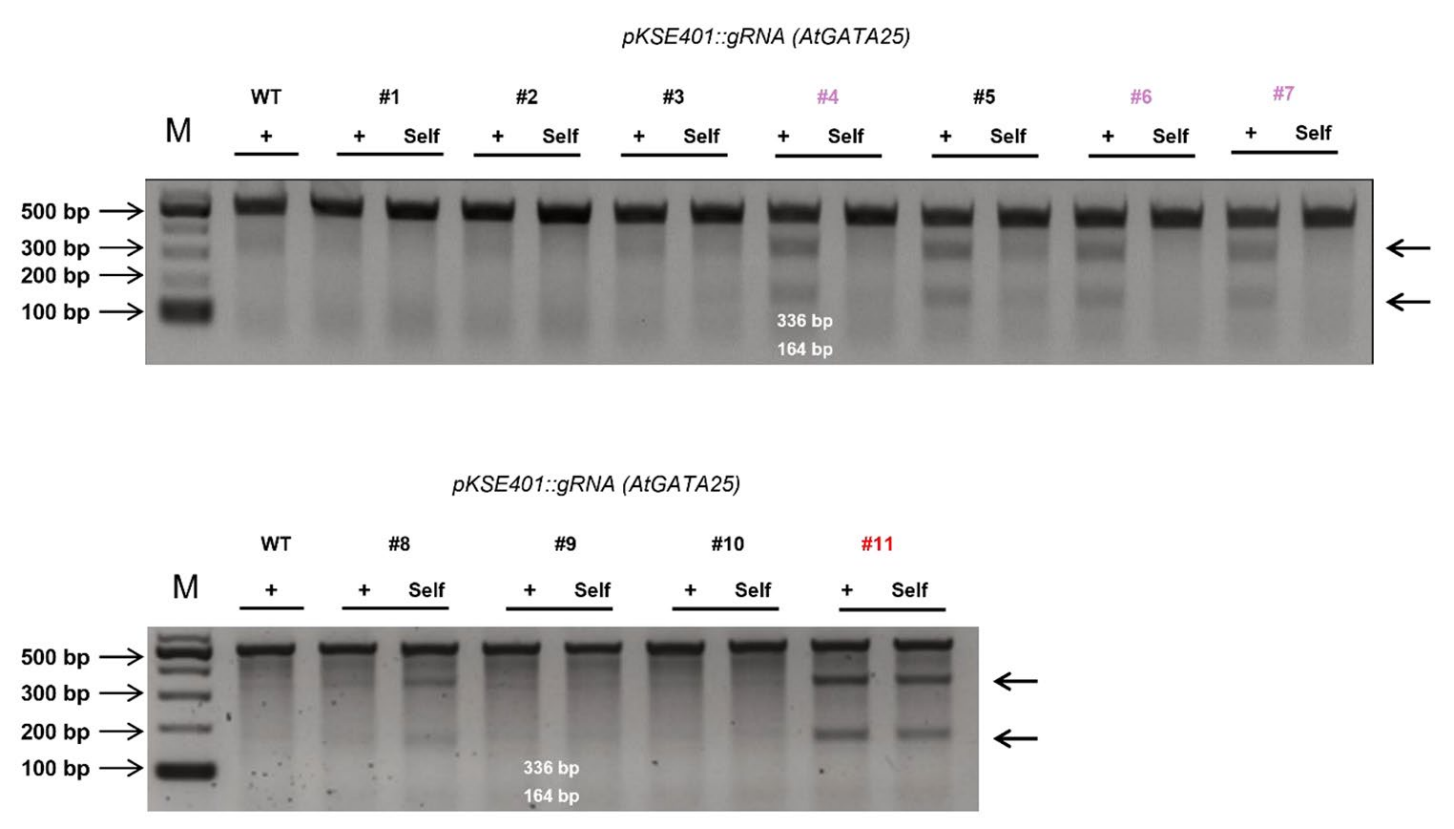
Mutation verification of *AtGATA25* with T7-endonuclease I (T7E1) assay. Detection of indels by a T7E1 assay in the endogenous *AtGATA25*. Cleavage products of the T7E1 assay are marked by black arrows. M, 1-kb ladder DNA marker; +, the result of performing T7E1 assay using the PCR product obtained by mixing equal amounts of the PCR product of the WT and PCR product of each mutant line; Self, the result of performing T7E1 assay using the PCR product obtained by mixing equal amounts of the PCR product of each mutant line

We used the CRISPR/Cas9 system to generate a loss-of-function mutation in *AtGATA25* in *Arabidopsis thaliana* Col-0. The oligonucleotides for the gRNA were synthesized and cloned into the pKSE401 binary vector. The binary vector pKSE401*-AtGATA25* was introduced into the *Arabidopsis thaliana* Col-0 through *Agrobacterium*-mediated transformation (Fig.1b). Thirty kanamycin-resistant transgenic plants were successfully generated. The T_2_ generation of transgenic plants was screened using kanamycin as a selection marker, and a single-copy cassette showed a Mendelian segregation ratio (3:1). Then, we selected 11 representative lines from kanamycin-resistant transgenic plants and confirmed mutagenesis using the T7E1 assay (Fig.2). The bands cleaved by T7 endonuclease I indicate that the on-target region (*AtGATA25*) of WT and the on-target region of the transgenic plants were mismatched by insertion or deletion of the mutation (indel). The cleavage bands were visible in six transgenic lines (#4, #5, #6, #7, #8, and #11). In particular, we developed a rapid method for the efficient identification of homozygous mutants using the T7E1 assay through self-hybridization in on-target region of transgenic plants. In this study, six homozygous mutants were identified.

### Identification of the mutation in the coding sequence of *AtGATA25*

**Fig. 3:**
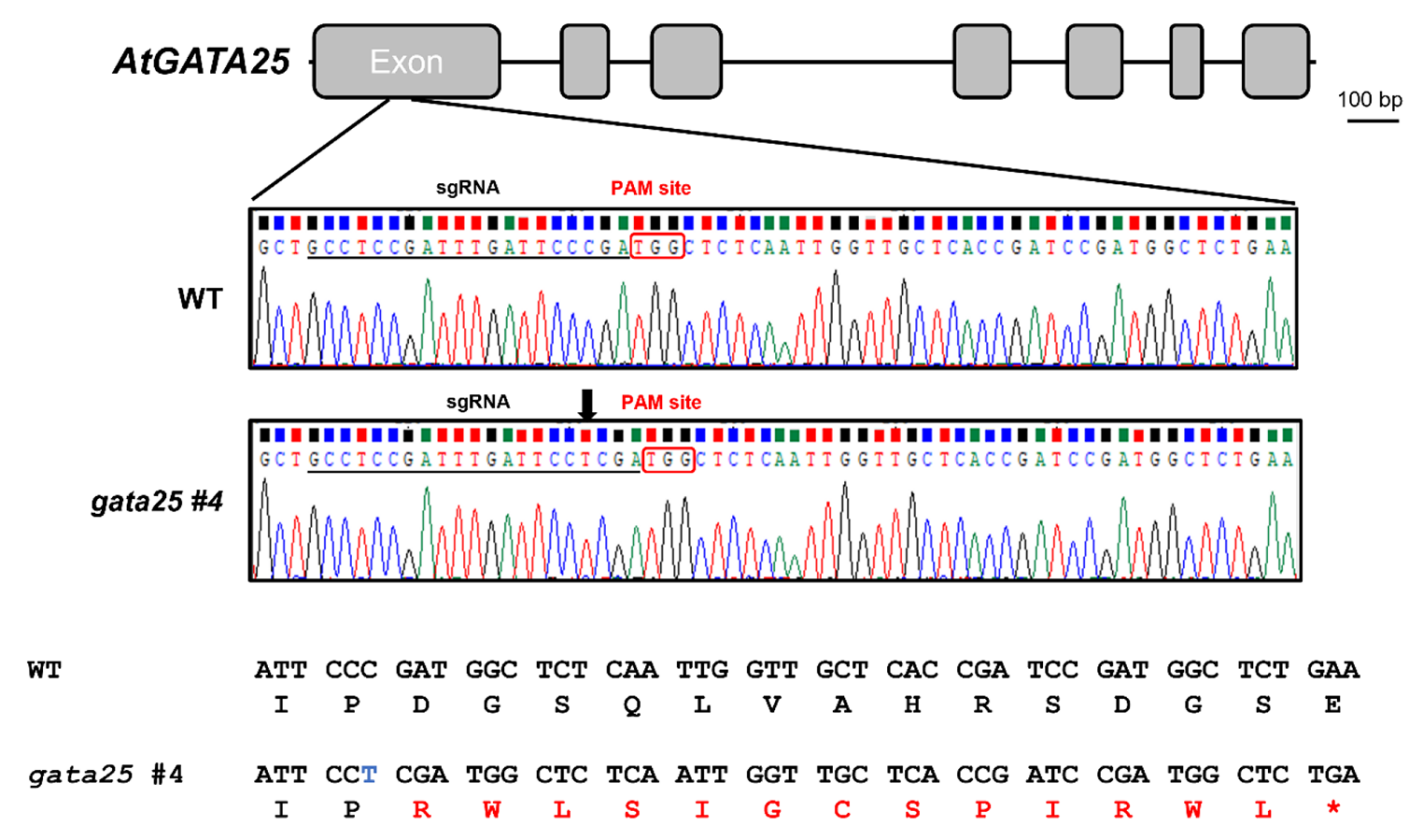
Targeted mutagenesis of *AtGATA25* using CRISPR/Cas9 system. Schematic representation of *AtGATA25*. Sanger sequencing analysis of mutation patterns in target sites. The gRNA-targeted DNA are underlined and the protospacer adjacent motif (PAM) is in the red box. Inserted nucleotide is depicted by the vertical arrow. Black line, intron; Gray box, exon; *, stop codon; Blue letter means inserted nucleotide; Red letter means changed amino acid; Scale bar, 100 bp

To accurately identify the mutated sequence induced by the CRISPR/Cas9 system, we extracted genomic DNA from the leaves of six homozygous transgenic plants. Then, the periphery of the on-target region in each genomic DNA sample was amplified to 500 bp using PCR. The PCR products were characterized by the Sanger sequencing. We considered whether multiple PCR products were generated by error accumulation during PCR amplification for accurate sequencing. The Sanger sequencing result would not confirm the nucleotide sequence due to a miscellaneous peak if multiple PCR products were generated. Therefore, to prevent the production of multiple PCR products, a high-fidelity PrimeSTAR HS DNA polymerase was used. The Sanger sequencing result confirmed that Chromas software clearly presented the nucleotide peaks (Fig.3). The automated annotated DNA sequencer was displayed as a four-color chromatogram showing the best guess data when interpreting the sequencing results data. However, these computer programs are prone to errors and must be manually re-checked to interpret the underlying data.

As a result of the Sanger sequencing, it was confirmed that one nucleotide (T, thymine) was equally inserted in the six homozygous transgenic plants. This frameshift mutation caused by one nucleotide insertion was predicted to result in an early stop codon in the first exon. Accordingly, we confirmed that the number of amino acids in the *AtGATA25* (309 amino acids) mutant was changed. In the *gata25* mutant, the aspartic acid (D) at position 58 was replaced with arginine (R). Thus, the AtGATA25 protein had premature amino acid termination due to nucleotide insertion, suggesting loss-of-function enzymatic activity.

### AtGATA25 regulates hypocotyl elongation

**Fig. 4:**
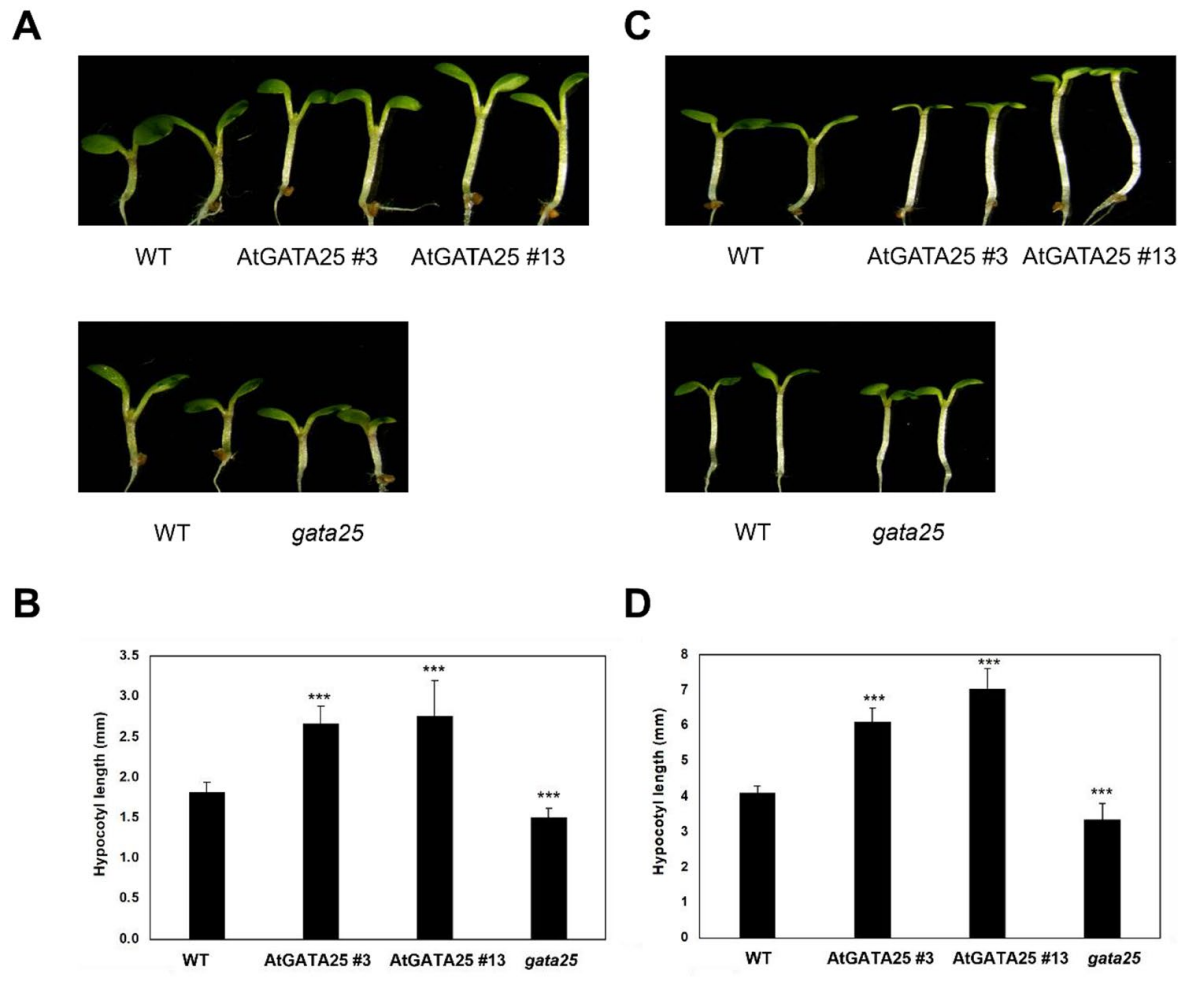
Hypocotyl phenotypes and length of 5-day-old *Arabidopsis thaliana* Col-0 (WT), AtGATA25 #3, AtGATA25 #13, and *gata25* seedlings grown in LD (**A** and **B**) or SD (**C** and **D**) conditions. Error bars represent the standard deviation (SD); n **≥** 13. Asterisks indicated statistically significant differences as determined by Student’s *t*-test (^***^p < 0.001). The graphs depict one of three experiments

A previous study demonstrated that AtGATA25 (*ZIM*) results in hypocotyl and petiole cell elongation [21]. To characterize whether AtGATA25 might play a role in the photoperiodic response of hypocotyl elongation, we grew *AtGATA25* overexpression transgenic lines and *gata25* mutants in LD and SD conditions. Two independent *AtGATA25* overexpression transgenic lines displayed longer seedling hypocotyls in LD compared to the WT. In contrast, the *gata25* mutant exhibited shorter seedling hypocotyls than WT (Fig.4a). Statistical analysis showed that seedling hypocotyl length in WT, *AtGATA25*-OX #3, *AtGATA25*-OX #13, *gata25* mutant grown in LD were 1.8 mm, 2.7 mm, 2.8 mm, and 1.5 mm on average, respectively (Fig.4b). As expected, AtGATA25 accelerated hypocotyl elongation in LD. Interestingly, seedlings of two independent *AtGATA25* overexpression transgenic lines displayed drastically increased hypocotyl length in SD compared to the WT. In addition, the seedlings of the *gata25* mutant displayed a drastically decreased hypocotyl length in SD compared to the WT (Fig.4c). Statistical analysis showed that seedling hypocotyl length in WT, *AtGATA25*-OX #3, *AtGATA25*-OX #13, and *gata25* mutant grown in SD was 4.1 mm, 6.1 mm, 7.1 mm, and 3.3 mm on average, respectively (Fig.4d). These results indicated that AtGATA25 promotes hypocotyl elongation regardless of LD or SD.

### AtGATA25 regulates the PIF4-mediated hypocotyl elongation

**Fig. 5:**
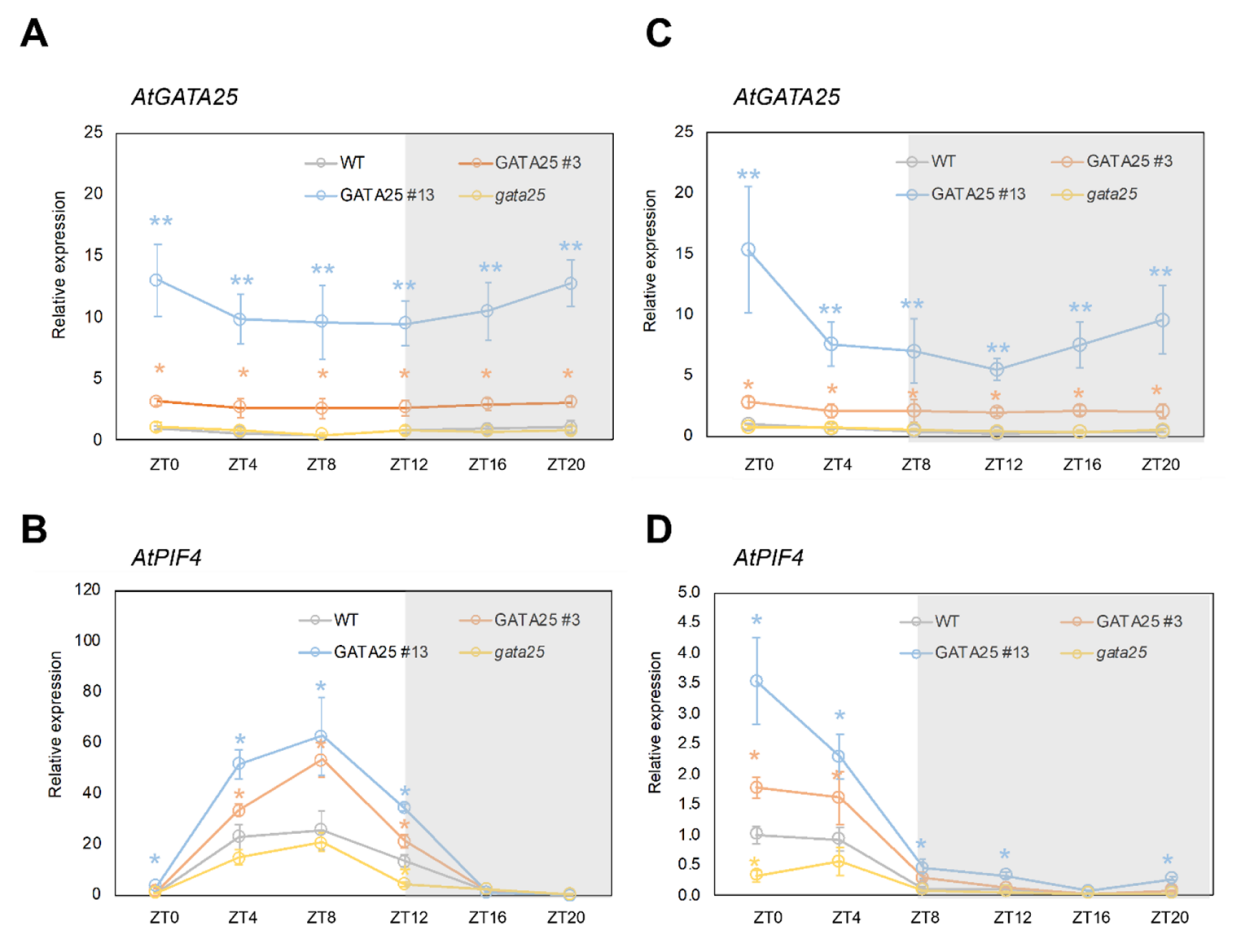
Examination of the diurnal expression profiles of an *AtGATA25* and *AtPIF4*. Quantitative real-time PCR analysis of *AtGATA25* and *AtPIF4* transcript levels. 5-day-old seedlings were grown in LD (**A** and **B**) and SD (**C** and **D**) conditions. mRNA samples were prepared at 4 h intervals. Expression levels relative to the peak position (each WT in ZT0 set to 1.0) are presented as mean values ± standard deviation (SD) of three biological replicates Asterisks indicated statistically significant differences compared to WT as determined by Student’s *t*-test (^*^p < 0.05, ^**^p < 0.01)

We hypothesized that the promotion of hypocotyl elongation is closely related to *PIF4*. Therefore, we investigated whether AtGATA25 activated the transcriptional activation of *PIF4* transcription levels in vivo. Given that *PIF4* transcription levels oscillate differently in LD and SD conditions, we analyzed the *PIF4* transcription levels at a different circadian time in the WT, *AtGATA25*-OX, and *gata25* mutants. First, we investigated that *AtGATA25* transcription levels in the WT, *AtGATA25*-OX, and *gata25* mutants. *AtGATA25*-OX lines maintained high levels of *AtGATA25* transcription throughout the day compared to the WT in LD and SD (Fig.5a, c). The *gata25* mutant showed a diurnal expression pattern similar to that of the WT in LD and SD. These results suggest that the premature stop codon of the *AtGATA25* gene was induced by the CRISPR/Cas9-mediated gene editing system, but it had no effect on the promoter region affecting the transcription level. Therefore, it is thought that the *gata25* mutant exhibits an expression pattern similar to that of the WT. The *PIF4* transcription levels in both the *AtGATA25*-OX lines increased in the morning regardless of LD and SD (Fig.5b, d). Particularly, the *PIF4* transcription levels were observed to significantly increased in SD compared to the WT throughout the circadian cycle except for ZT16. Contrastingly, the *gata25* mutant showed reduced *PIF4* transcription levels in LD and SD during the day, and significantly decreased the *PIF4* transcription levels in ZT12 and ZT0 in LD and SD, respectively. These results may indicated that the accumulation of *PIF4* transcripts induced by AtGATA25 resulted in accelerated hypocotyl elongation. Notably, accelerated hypocotyl elongation in SD at normal temperature compared to LD is consistent with a previous study in which PIF4 activity was absent during the day, and PIF4 activity appeared at night [44].

### AtGATA25 transcription factor binds to the *PIF4 *promoter

**Fig. 6:**
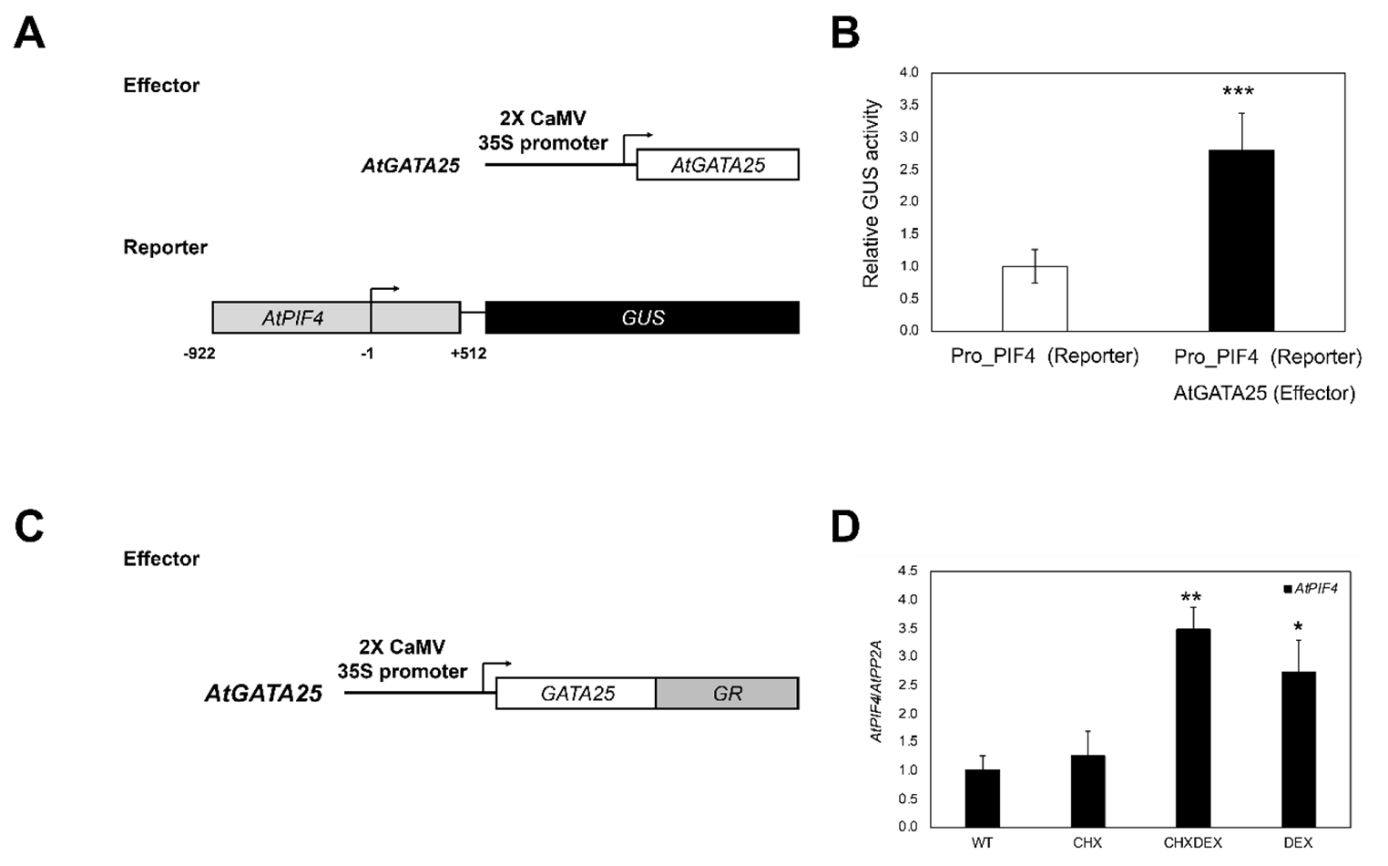
*AtGATA25* binds to the *PIF4* promoter and activates the expression of *PIF4*. **A** Schematic diagram of the effector and reporter constructs used. *AtGATA25* (effector) and GUS reporter gene driven by the *PIF4* promoter (reporter) were co-expressed in Arabidopsis leaf protoplasts. **B** Transcriptional activation analysis showing that the AtGATA25 activates the promoter of *PIF4*. The activity of the GUS in the reporter construct transfected protoplasts with no effector was used as a control. **C** Schematic diagram of the *AtGATA25*-GR (effector) used in the transcriptional activation analysis. **D** AtGATA25 directly increases the *PIF4* transcription levels by DEX activation of AtGATA25-GR that occurred in the presence of CHX by performing the qRT-PCR. Error bars indicate the standard deviation (SD) of three biological replicates. Asterisks indicated statistically significant differences compared to control as determined by Student’s *t*-test ((^*^p < 0.05, ^**^p < 0.01, ^***^p < 0.001)

The AtGATA25 transcription factor is likely recruited to the DNA motif via the TGATAA or AGATAA sequence [21, 45]. We investigated whether the AtGATA25 transcription factor binds to the *PIF4* promoter. Based on the 1.5 kb native *PIF4* promoter sequence obtained from the TAIR, we found that the *PIF4* promoter contains a putative GATA binding motif (three TGATAA and five AGATAA sequences). We used a transcriptional activation analysis to test the possibility that the AtGATAT25 transcription factor activates transcription of *PIF4* (Fig.6). The 2Χ CaMV 35S promoter-driven *AtGATA25* expression construct (effector) and the 1.4 kb native *PIF4* promoter-driven *GUS* expression construct (reporter) were co-transfected in *Arabidopsis* mesophyll protoplasts (Fig.6a). As expected, when the *AtGATA25* effector driven by the 2Χ CaMV 35S promoter was expressed in *Arabidopsis* mesophyll protoplasts, GUS activity of the reporter with the *GUS* gene driven by the native *PIF4* promoter was significantly increased by 2.8-fold compared to the control, which transfected reporter in the *Arabidopsis* mesophyll protoplasts (Fig.6b). Taken together, these results were suggested that AtGATA25 binds to the *PIF4* promoter and induces the accumulation of *PIF4* transcripts, thereby promoting PIF4-mediated hypocotyl elongation.

To determine whether AtGATA25 directly activates the expression of *PIF4*, we used a steroid receptor-based inducible activation system [41]. This system uses the glucocorticoid receptor hormone-binding domain (GR) fused transcription factor that binds to heat shock protein 90 (HSP90) in the cytoplasm to form a cytoplasmic complex and treats DEX to allow the fusion protein to enter the nucleus. In this study, the 2Χ CaMV 35S promoter-driven *AtGATA25*-GR expression construct was transfected into *Arabidopsis* mesophyll protoplasts (Fig.6c). DEX-activated AtGATA25-GR increased *PIF4* transcription levels. In addition, AtGATA25-GR treated simultaneously with CHX and DEX induced the *PIF4* transcription levels compared to CHX alone. These results suggest that AtGATA25 regulates the transcription of *PIF4* gene by activating the *PIF4* promoter.

## Discussion

The CRISPR/Cas9-mediated gene editing system is very useful in plant cell biology because it provides a way to generate gene knockouts in plants [46]. In CRISPR/Cas9-mediated gene editing systems, CRISPR/Cas9-induced site-specific DNA double-stranded breaks (DSBs) are repaired by either the homology-directed repair (HDR) or non-homologous end-joining (NHEJ) pathways. Previous studies have shown that gene editing via the HDR pathway is exceptionally inefficient and is widely applied in a few higher plants [47]. Contrastingly, the error-prone NHEJ pathway has been reported to efficiently induce random mutagenesis in various plant species [48]. In this study, we attempted to efficiently generate an *AtGATA25* knockout *Arabidopsis thaliana* using the CRISPR/Cas9-mediated gene editing system of the NHEJ pathway. To increase the efficiency of the CRISPR/Cas9-mediated gene editing system, the correct *AtGATA25* sequence was confirmed (Fig.1, Supplementary Fig. S1). This result confirmed that it differed from the *AtGATA25* nucleotide sequence known in TAIR. This sequencing result is consistent with the amino acid sequence of a previous study confirming the transactivation assay of AtGATA25 (ZIM) [49]. Therefore, using the identified *AtGATA25* nucleotide sequence, a 20 bp gRNA was designed using the online software CRISPR-P (version 2.0; Supplementary Fig. S2). A novel strategy, modified from the conventional T7E1 assay, for the rapid and efficient selection of homozygous mutants was attempted. In the conventional T7E1 assay, the PCR product of the target gene of the WT and transgenic lines was hybridized and treated with T7 endonuclease I. However, in the modified T7E1 assay used in this study, the transgenic line was further self-hybridized with the PCR product of the target gene and treated with T7 endonuclease I (Fig.2). This modified T7E1 assay can be used to select transgenic lines where the mutant allele has the same mutant allele during meiosis. The type of mutation was observed by the Sanger sequencing of the transgenic line selected using the modified T7E1 assay (Fig.3). Consequently, we successfully generated a homozygous *gata25* mutant using the CRISPR/Cas9 gene editing system in *Arabidopsis thaliana*. To the best of our knowledge, this is the first study to investigate *AtGATA25* knockout in the plants.

The hypocotyl length of the *gata25* mutant was shorter than that of the WT regardless of LD and SD and the hypocotyl length of *AtGATA25*-OX was longer than that of the WT regardless of LD and SD (Fig.4). We also observed more pronounced hypocotyl length differences in SD than in LD. Therefore, we hypothesized that the mechanisms that regulate hypocotyl elongation are closely related to the post-integration of photoreceptor-mediated light signaling. A previous study found similar results related to hypocotyl elongation [50, 51]. PIF4 acts as a critical regulator of light signal transduction and is a key regulator of the thermoresponsive pathway. Additionally, PIF4 integrates with growth-regulating hormones, including auxins and gibberellins, to mediate the expression of a series of thermoresponsive genes. As a result of observing the expression pattern of *PIF4* in plants, *PIF4* expression was accumulated in *AtGATA25*-OX regardless of LD and SD in light. Particularly, the expression of *PIF4* gene can be predicted to increases between ZT20 and ZT24 (or ZT0) in LD. In addition, *PIF4* expression was observed to be accumulated in ZT8, ZT12, and ZT20 in *AtGATA25*-OX in SD. Contrastingly, *PIF4* expression was inhibited in the *gata25* mutant (Fig.5). This is based on the previous observation that post-translational expression of PIF4 is modulated by circadian gating mediated by the TOC1-PIF4 interaction [17], suggesting that long darkness in SD allows PIF4 to sufficiently induce expression of the downstream target gene. Accordingly, it may be explained that the hypocotyl length is more distinctly different in SD. These results were further supported by *PIF4* promoter activity analysis (Fig.6). Through transcriptional activation analysis using *Arabidopsis* protoplasts, it was confirmed that the AtGATA25 transcription factor binds to the *PIF4* promoter and induces the expression of *PIF4*. This strongly correlates with the higher expression of *AtGATA25* at night during the circadian rhythm [22] and the higher expression of *PIF4* in *AtGATA25*-OX plants at night. However, although the *AtGATA25* gene was overexpressed in LD and SD, it was confirmed that the transcription level of *PIF4* differed depending on the day/night cycle. These results may suggest that AtGATA25 protein levels change throughout the day. Therefore, it is thought necessary to confirm the changes in the AtGATA25 protein levels through additional experiments. Additionally, PIF4 has been reported to be a critical regulatory factor for the temperature-sensing pathway interaction via the photoperiodic pathway [52–54]. Accordingly, further studies on thermomorphogenesis may clearly elucidate the regulatory mechanisms regulating PIF4-mediated hypocotyl elongation by AtGATA25.

## Conclusion

The hypocotyl elongation mechanism of *Arabidopsis thaliana* seedlings was confirmed using the CRISPR/Cas9-mediated *AtGATA25* mutant and the *AtGATA25* overexpressing transgenic plant was used in our experiments. The hypocotyl growth rate of each plant was confirmed, and the expression of *PIF4* regulated in the circadian clock was divided into LD and SD to examine the *PIF4* expression. Our results suggest that AtGATA25 induces the expression of *PIF4* and is a novel model for hypocotyl elongation. These findings provide an understanding of novel mechanisms of hypocotyl elongation in plants.

## Electronic supplementary material

Supplementary Material
