# Supplementary material for: CRISPR/Cas9-mediated AtGATA25 mutant represents a novel model for regulating hypocotyl elongation in Arabidopsis thaliana
Source: Mol Biol Rep. 2022 Oct 27;50(1):31–41. doi: 10.1007/s11033-022-07926-9 (PMC9884261; doi:10.1007/s11033-022-07926-9)
Supplement: Supplementary file 1 — Supplementary Material [file 11033_2022_7926_MOESM1_ESM.doc]

**
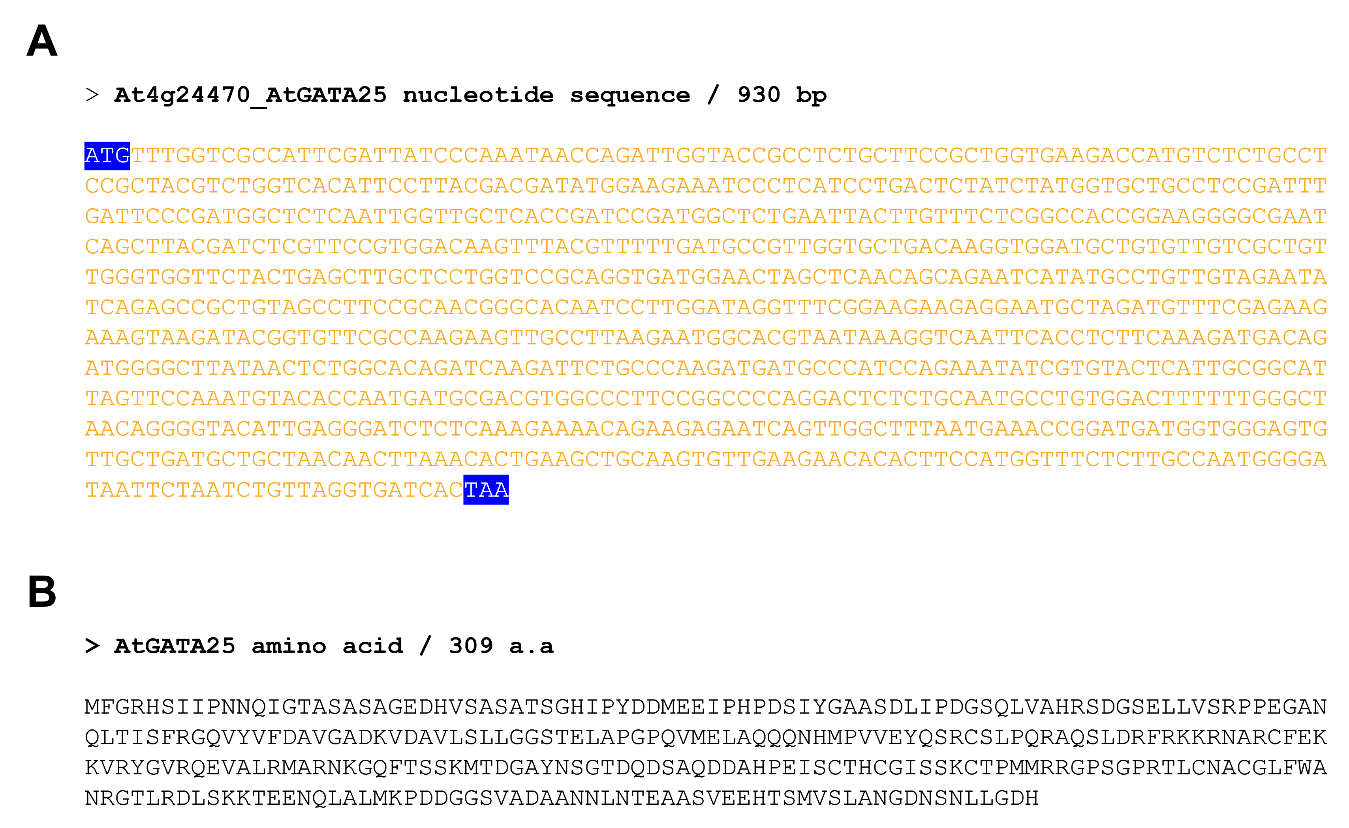
**

**Supplementary Fig. S1** Sequence analysis of *AtGATA25* gene. **A** *AtGATA25* cDNA sequence obtained from *Arabidopsis thaliana* Col-0. **B** Protein sequence deduced from the *AtGATA25* cDNA sequences

**
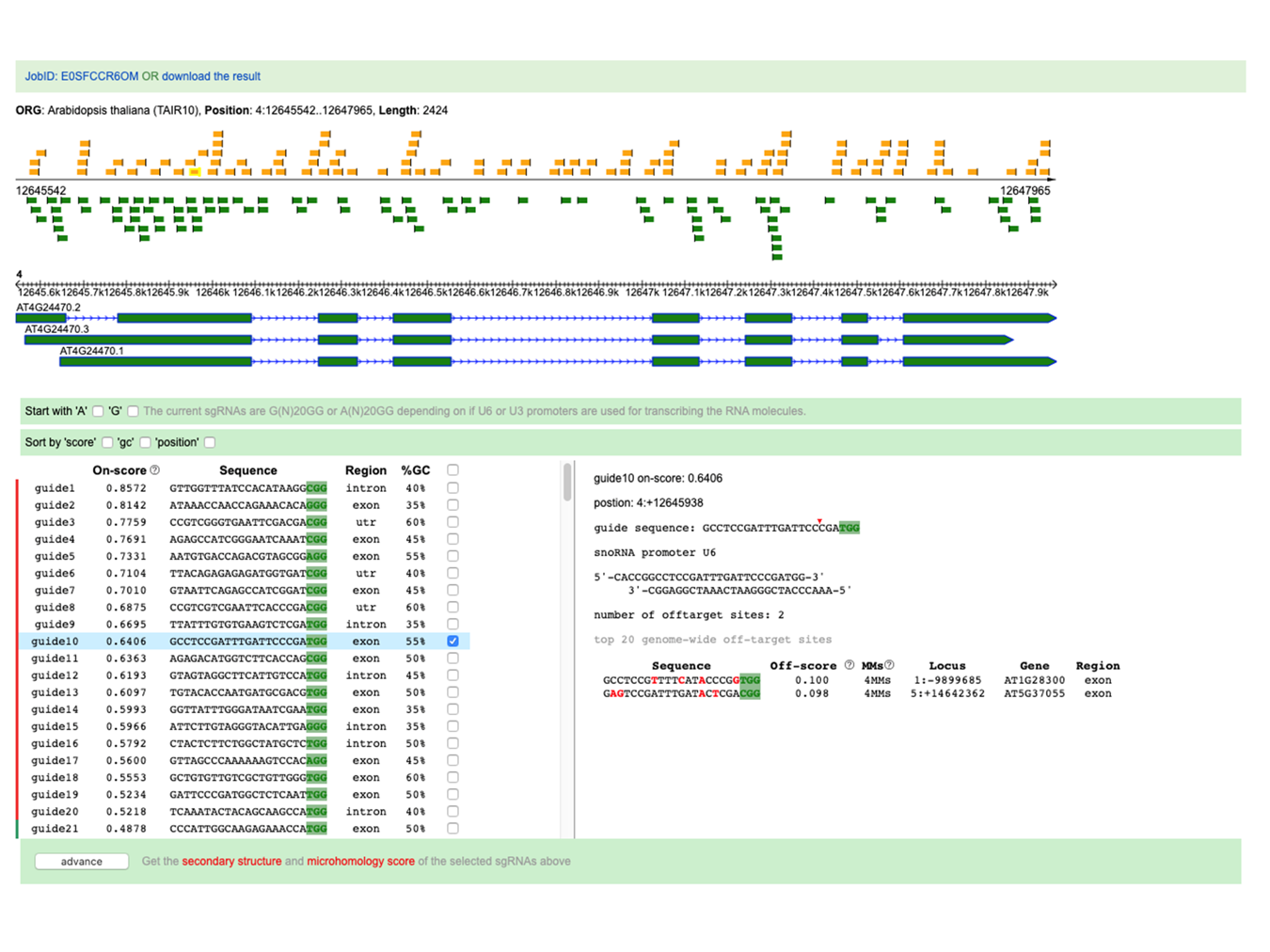
Supplementary Fig. S2** gRNA design result of AtGATA25 from *Arabidopsis thaliana* using CRISPR-P 2.0

**Supplementary Table. S1** List of primers used in this study

**
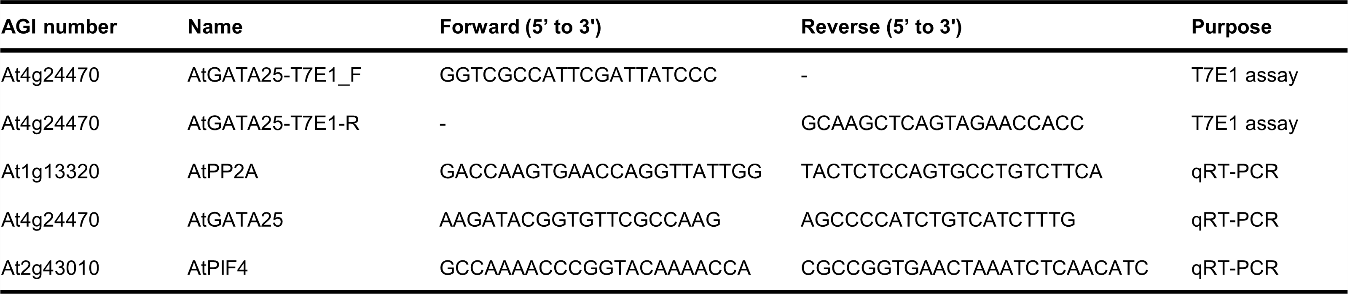
**
